# The use of ciprofloxacin and fluconazole in Italian neonatal intensive care units: a nationwide survey

**DOI:** 10.1186/1471-2431-13-5

**Published:** 2013-01-07

**Authors:** Chiara Pandolfini, Sequi Marco, Manzoni Paolo, Bonati Maurizio

**Affiliations:** 1 Laboratory for Mother and Child Health, Department of Public Health “Mario Negri” Pharmacological Research Institute, Milan, Italy; 2 Neonatology and Hospital Neonatal Intensive Care Unit, Azienda Ospedaliera Regina Margherita – Sant’Anna, Turin, Italy

**Keywords:** Health care surveys, Intensive care units, Neonatal, Italy, Ciprofloxacin, Fluconazole, Practice guidelines as topic

## Abstract

**Background:**

Treatment and prophylaxis of sepsis in very low birth weight neonates is a matter of concern and research is being undertaken with the aim to give rise to shared approaches based on solid evidence. As part of a European initiative, a survey was set up to describe the use of two drugs in this area. The Italian national practices concerning neonatal sepsis, as well as calls for related guidance, are described.

**Methods:**

A standardized and previously tested questionnaire was submitted online to all Italian level III NICUs. A 5-point Likert scale was used to analyze attitudinal replies. Categorical variables were compared by χ2 analysis and 2-tailed P-values are reported.

**Results:**

Data was provided by 38 Italian NICUs (36% of the country’s level III centers), 53% of which have 1–10 cases of bacterial sepsis monthly and 90% a prevalence of <1% fungal infections. Ciprofloxacin and fluconazole treatment for neonatal sepsis are scantly used in Italian NICUs (13% and 45%, respectively). Major concerns are related to the safety of ciprofloxacin and the efficacy of fluconazole. On the contrary, prophylaxis of fungal infections is a routine approach in many Italian NICUs. The use of both ciprofloxacin and fluconazole is characterized by a large inter-NICU variability in dose and scheme of use. The lack of adequate, shared evidence is a common consideration made by the survey participants.

**Conclusions:**

Common approaches are needed to standardize and update a national drug strategy for the prevention and treatment of sepsis in very low birth weight newborns. This can be achieved through collaborative initiatives aimed at setting up guidelines, based on available data, and multicenter trials to produce new evidence that will address the knowledge gaps.

## Background

Sepsis in neonates can be caused by bacterial or fungal microorganisms and is associated with significant morbidity and mortality. Up to one fourth of very-low-birth weight infants (VLBW; <1500 g at birth) develop hospital-acquired infections.

Studies have assessed the use prophylactic measures to prevent infections, but data on treatment and prophylaxis is lacking [1-3]. Empirical antibiotic therapy is often used in NICUs [4], although it can lead to unnecessary exposure to antibiotics, can increase the risk of neonatal death, and can cause selective pressure for antibiotic resistance, making it fundamental to employ only the best possible options and only when necessary [5-9]. Efficacy data on antifungals in the treatment of invasive fungal infections in preterm infants is lacking and more data on their use in the prevention of such infections are needed as well [10,11], although partial guidelines do exist [12-14].

Italy has been playing a lead role in the investigation of neonatal infections, also as a result of the activities of a collaborative network, the GSIN (Gruppo di Studio delle Infezioni Neonatali), specifically dedicated to performing studies in this area [15-17].

A European project called Treat Infections in Neonates (TINN), coordinated by the French National Institute for Health and Medical Research in France and the University of Nottingham in the UK, with the aim to evaluate the safety and efficacy of ciprofloxacin and fluconazole in preterm and term neonates, is currently running [18]. The project involves partners in 7 countries and is supported by the European Commission. Ciprofloxacin and fluconazole are being studied because they are present in the European Medicines Agency’s priority list of therapeutic areas that need specific drug evaluation in preterm and term neonates [19]. As part of the TINN project, a European survey on the use of ciprofloxacin for treatment, and fluconazole for treatment and prophylaxis, of invasive infections in neonates was carried out [20,21]. The main objective was to describe the current use of the two drugs in NICUs in order to better orient the efforts of the entire TINN project towards the creation of necessary knowledge on the optimal use of these drugs.

Of all European NICUs that participated in the survey, Italy was the most represented country, both in terms of number of NICUs and homogeneity of their profile (all level III units). In the present paper, we focus on the Italian findings of the survey in order to describe the national practices concerning neonatal sepsis.

## Methods

All Italian level III NICUs were contacted via email between December 2009 and May 2010 with both a letter of invitation to participate in the survey and a commitment to provide all participants with a report of the survey’s results.

A questionnaire collecting information on the characteristics of each unit, its use of ciprofloxacin and fluconazole, and its involvement in clinical research was created by the TINN partners, based in part on a US survey [22]. The survey was tested by a group of partners, and an online version for input was set up and sent to each Italian NICU.

A number of questions assessing the factors that influence the NICU’s policies regarding ciprofloxacin and fluconazole were included, and an additional email requesting missing data was sent in June 2010 to NICUs who had almost completed all the questions necessary for their records to be considered in the analysis.

### Statistical analysis

A methodology similar to that employed in the US survey by Burwell [22] was used in order to enhance comparison of the respective results. Another, more recent study from UK and Ireland also used a similar questionnaire, but it was administered to individual physicians so data does not refer to individual intensive care units [23]. The answers to the questions included in the questionnaire were measured on a 5-point Likert scale (1: least important, 5: most important). The mean and 95% confidence intervals were calculated for the 5-point Likert scale responses. Since most of the means were between 3 and 4, the responses were dichotomized and those ≥ 4 were categorized as important and those ≤ 3 as less important. Respondents were grouped into those who used ciprofloxacin and those who did not, and those who used fluconazole and those who did not. Categorical variables were compared by χ2 analysis and *P* values are 2-tailed. The survey data collected was managed using Microsoft Access and analyzed using SAS (Vers. 9.13, SAS Institute, Inc. Cary, NC).

The “Mario Negri” Institutional review board ruled the study exempt from ethical approval.

## Results

Data were obtained from 38 Italian NICUs, 36% (38/105) of the country’s level III centers. Over half (53%) the NICUs have 1–10 cases of bacterial sepsis monthly, while 42% have less than 1. The reported prevalence of fungal infections is <1% in 50% of the NICUs and is ≤ 5% in 90%.

In terms of geographical distribution, almost half (47%) of the NICUs that participated are located in northern Italy, while 19% are in central and 34% in southern Italy. The proportion of participating NICUs compared to the number of NICUs present in each of the three geographical areas is similar; no statistically significant difference was found (p=0.36). The data would therefore seem to be representative of the regional distribution of NICUs.

Most of the NICUs (71%) have a standard written protocol on antibiotic treatment in case of suspected bacterial sepsis, 82% have one on routine antifungal prophylaxis, and 70% have one on antifungal treatment. 47% of NICUs have protocols covering all three conditions.

### Ciprofloxacin treatment

Only 5 NICUs (13%) ever use ciprofloxacin. The indications for initiating treatment vary between NICUs [Table 1]. All 5 administer the drug intravenously, but at different dosages: three administer an average dose of 20 mg/kg/day (at 12 hour intervals), while the other two administer 10 mg/kg/day (one at 8 hour intervals and the other at 12). Concerning the average duration of treatment, only one center replied, reporting 10 days.

**Table 1:** Indications for initiation of treatment or prophylaxis with the two drugs

**Ciprofloxacin treatment (5 NICUs)**	**N. NICUs**
First line therapy (even in association) of:	
Culture-proven sepsis due to multi-drug resistant organisms that are sensitive to ciprofloxacin	5 (100%)
Sepsis resulting from a suspected multi-drug resistant strain infection	1
Sepsis resistant to first line empirical antibiotic therapy (other than ciprofloxacin)	1
* Severe* sepsis resistant to first line empirical antibiotic therapy (other than ciprofloxacin)	1
**Fluconazole prophylaxis (30 NICUs)**	
Birth gestational age	21 (70%)
<26 weeks	2
<28 weeks	10
<30 weeks	7
other	2
Birth weight	27 (90%)
<750 g	1
<1000g	13
<1500	13
Neonate receiving antibiotics for a certain number of days	14 (47%)
>2 days	6
>7 days	5
>14 days	3
Presence of a central venous catheter (CVC)	22 (73%)
Peripherally inserted CVC	18
Umbilical venous CVC	15
Only CVC used >7 days	7
Only CVC used >14 days	1
Surgically inserted CVC	5
Endotracheal intubation	10 (33%)
Only if intubated >7 days	8
Only if intubated >14 days	0
Total parenteral nutrition (TPN)	14 (47%)
Only if TPN used >7 days	11
Only if TPN used >14 days	1
Abdominal surgery	12 (40%)
Abdominal disease	7 (23%)
Necrotizing enterocolitis	4
Focal bowel perforation	3
Gastroschisis	1
Omphalocele	0
Antibiotics being used	5 (17%)
Only if >7 days	5
Only if >14 days	0
Colonization status	11 (37%)
Scheduled surveillance cultures+/− other cultures	7
Determined by clinical features	4
**Fluconazole treatment (17 NICUs)**	
Sepsis with identification of Candida spp. in cultures of central samples (blood, cerebrospinal fluid, …)	15 (88%)
Sepsis with documented fungal colonization	12 (71%)
Sepsis resistant to first line empirical antibiotic therapy	7 (41%)
Severe sepsis	1 (6%)

Note: some answers were missing so sum is not always equal to number of NICUs replying.

Among the factors influencing the decision to use ciprofloxacin or not, the two ranked most important were uncertainty about the safety of the drug and the fact that ciprofloxacin should be reserved for infections with multi-drug resistant micro-organisms (average rating of each factor, from 1 to 5: 4,2).When NICUs using ciprofloxacin were compared with those not using it, the numbers were unbalanced, but they seemed to show no significant difference in importance attributed to the listed factors [See Additional file 1 for data].

No guidelines on the use of ciprofloxacin for sepsis were found. The British national Formulary for Children (BNF-C) [24] recommends the use of other agents in treating neonatal bacteremia and mentions that ciprofloxacin is used in cases of septicemia caused by multiresistant organisms, but does not address the drug directly.

### Fluconazole prophylaxis

Overall, 30 NICUs (79%) declare using fluconazole for prophylaxis. No significant correlation was found between use of fluconazole prophylaxis and prevalence of fungal infections in the NICUs (Fisher's exact test P-value 1.0000).

In the centers that use fluconazole for prophylaxis, birth weight is an important variable guiding the decision to employ prophylaxis for 90% of them. Of these centers, 48% use <1500 g as a cutoff, 48% use <1000 g, and the remaining NICU uses <750 g as cutoff. There was wide variation in the indications for initiating prophylaxis between NICUs [Table 1].

Differences were found between the 30 NICUs in the dosage schemes used as well. The majority (76%) administer an average dose of 3 mg/kg, while the rest administer 6 mg/kg. Of those who use 3 mg/kg, a little more than 50% administer it every 72 hours, 36% every 48 hours. Of the 7 centers who use 6 mg/kg, 4 administer it every 72 hours and the rest every 48, 24, or “other” hours (1 each). Most NICUs (89%) administer the drug intravenously, while the rest administer it orally.

Concerning the duration and withdrawal of prophylaxis, almost half (47%) reported having a fixed average duration of prophylaxis, which ranged from 10 to 45 days (median 30). Few centers reported using corrected gestational age or target weight reached as parameters (2 and 1 NICUs, respectively), while 12 (40%) reported unavailability of an IV route as a criterium. In cases of confirmed fungal infection in neonates receiving prophylactic fluconazole, 14 (47%) of the NICUs report using liposomal amphotericin B, 2 report amphotericine, 4 fluconazole at increased, therapeutic dosages, 2 flucytosine, and 1 caspofungin (in cases of resistance).

NICUs who did not use fluconazole prophylaxis were significantly more likely to be concerned about the existing uncertainty regarding the drug’s safety in newborns (p<0.01). The need for a statement by pediatric societies in support of the routine use of fluconazole for prophylaxis and the need for additional efficacy studies were also considered important factors (average importance, from 1 to 5: 3.5 and 3.3, respectively) [See Additional file 1 for data].

### Fluconazole treatment

Almost half (17) of NICUs administer fluconazole for treatment. Of the centers with a protocol (26/38), over half (56%) don’t use fluconazole. Overall, 12 NICUs use fluconazole for both prophylaxis and treatment. Most of the NICUs (15/17) use fluconazole in cases of neonatal sepsis with identification of *Candida spp* in cultures of central samples, although there was some variation in indications for starting treatment also for fluconazole [Table 1].

Fluconazole treatment schemes between NICUs were also found to vary. Dosages ranged from 3 to 12 mg/kg/daily, considering the different average single doses and the intervals applied between administrations. Most of the NICUs (15/17) administer fluconazole intravenously. The average duration of treatment ranges from 10 to 30 days. The current first line antifungal, other than fluconazole, used in NICUs is liposomal amphotericin B in 8 centers and amphotericin B in 2.

Current guidelines [13] on treatment of neonatal candidiasis recommend amphotericin B or, as a reasonable alternative, fluconazole at a dosage of 12 mg/kg daily for 3 weeks. Of the 17 NICUS that use fluconazole, 5 administer it at the recommended dosage, and for more or less the recommended length of therapy (3 administer it for 3 weeks, 1 for 28 days, and 1 for 15–21 days). All the rest administer lower dosages, either because of a lower unit dose or because the same dose is given less frequently. When transformed into daily doses: 9 NICUs give 6 mg/kg/day, 1 gives 4 mg/kg/day, 1 gives 3 mg/kg/day, and 1 does not specify frequency.

The factors potentially influencing the NICUs’ choice to use fluconazole for treatment were not considered very important by the NICUs. In fact, the highest average importance attributed was 3.3 and concerned two factors: the need for a statement by pediatric societies supporting the selection of the drug and the need for additional efficacy studies. No significant differences were found in the importance given to the different factors by NICUs who use the drug and those who don’t [See Additional file 1 for data].

## Discussion

Although all Italian level III NICUs were invited to join the survey, it is likely that those that participated are more active in research and/or are more interested in self-audit and improvement of a unanimously acknowledged clinical care. Nevertheless, a third of all the Italian NICUs participated and represent the north, center, and south of Italy. Besides the geographic distribution, the estimated rate of admission of newborns <32 weeks’ gestational age in participating NICUs (0.85%) is close to that reported in Italy (0.9%) [25] and also supports the representativeness of collected data at the national level. The wide variation in dosages used does not seem to depend on area of the country and, in any case, highlights the need for national, or better international, shared guidelines incorporating current knowledge on the treatment of neonatal sepsis.

### Ciprofloxacin treatment

Almost three quarters of Italian NICUs have a standard written protocol regarding antibiotic treatment for suspected sepsis, and very few use ciprofloxacin. The Italian data was in line with European data [20] in terms of limited use of ciprofloxacin and concern about bacterial resistance and drug safety. Concern about antibiotic resistance, however, was not enough to dissuade centres from using the drug. The ciprofloxacin dosages used varied, but the NICUs using this drug were few so no conclusions can be made.

### Fluconazole use

Fluconazole prophylaxis has been found to reduce the incidence of invasive fungal infection in VLBW infants, and recent data on long term outcomes and antifungal resistance seem positive so far [26,27]. This study’s data show that, in Italy, a majority of NICUs (79%) use fluconazole prophylaxis, a rate much higher than that found in all the European NICUs participating in the survey [20] and in the UK/Ireland and US studies [22,23]. Rates of prophylaxis in different studies may be based on incidence, but rates in infants at the lower gestational ages are high in all studies. No correlation was found between reported prevalence of fungal infections in the NICUs and their choice to use fluconazole prophylaxis. The reported incidence in the survey fits within the range reported by other studies [28], although methodologies in data collection differ and direct comparison with incidence data is not possible. Incidence rates of fungal infection differ between studies, from 2,6 to 9% [29,30]. A large part of the variation in rates is due to the number of extremely low birth weight infants admitted at each NICU [31,32], but may also reflect differences in clinical practices [33].

There was variation between the centers concerning the indications for initiating fluconazole prophylaxis, but birth weight, presence of a central venous catheter, and gestational age were considered the most important and these data are similar to those found by Burwell et al [22]. The guidelines addressing prophylaxis [12,13] only take into account birth weight and rate of disease in hospital units as the factors on which to base prophylaxis initiation, while other factors should also be considered [34]. Guidelines use birth weight as an indicator, for example, but gestational age has a more linear relationship with invasive *candida* infection [35]. Furthermore, the guidelines should be updated to reflect the finding that fluconazole prophylaxis can reduce incidence even in NICUs with a low rate of invasive *candida* infection [34]. It has been shown that implementing a guideline that includes specific criteria for identifying high-risk VLBW infants reduced invasive fungal sepsis rates without evidence of fluconazole resistance emerging [36]. The possibility of resistance emerging, as well as insufficient safety data, make the creation of standard, more accurate guidelines identifying newborns at the highest risk of acquiring fungal infection, and therefore candidates for prophylaxis, a high priority.

The main guidelines on candidiasis [13] suggest considering fluconazole prophylaxis in neonates with birth weights <1000 g, and at a dosage of 3 mg/kg iv every 72 hours for the first 2 weeks of life, every 48 hours during the third and fourth weeks, and daily in the fifth and sixth weeks of life. The other cited guidelines [14] offer limited information on prophylaxis, but the initial dose is similar. Dosage data from the NICUs refers to a single dose only, so a comparison with the dosage change over time, as specified in the main guidelines, is not possible, however, 38% of the NICUs report using the same dosage and route as the guidelines, i.e. 3 mg/kg every 72 hours intravenously. Only one center followed the guidelines completely, considering also duration of prophylaxis. The US survey did not assess dose, and data from the UK/Ireland study, which is not directly comparable because it involved individual physicians, had almost half the responses reporting the use of 3 mg/kg and almost one fourth 6 mg/kg, compared to three fourths using the lower dose in the Italian data. Studies have been performed to test less intensive dose regimens, including twice weekly dosing [17,37], and their results were encouraging, but additional trials are needed to identify the most appropriate dosing regimen [10].

Significant concern about safety in using fluconazole for prophylaxis resulted from the data. This differs from the US data [22], which revealed concern especially about resistance, and the UK/Ireland data [23], which emphasized the importance or the perceived level of invasive candidiasis in the unit. Even when compared with the data from all the European NICUs surveyed [20], showing that those that don’t use prophylaxis were more likely to be concerned about both resistance and the lack of a statement by pediatric societies promoting routine use in a subset of newborns, concerns seem to differ. To reduce the possibility of resistance in cases of fungal infection in infants following fluconazole prophylaxis, treatment with a different antifungal than fluconazole should be initiated [34]. Only 19/30 of the NICUs reported using a drug other than fluconazole in cases of confirmed fungal infection in patients under fluconazole prophylaxis. The guidelines should address this issue specifically.

Half of the NICUs use fluconazole for treatment of fungal sepsis, and only about one third follow the guideline recommendations on dosage, while the rest use lower dosages. Concerning the indications for starting treatment with fluconazole, the majority of NICUs that use the drug follow the guidelines in that they initiate treatment when sterile body fluids test positive for Candida spp. There did not seem to be general concern over safety, but some interest was expressed for both the need for an official statement supporting the choice of fluconazole and for more efficacy studies. These, however, did not seem to be valid indicators in the choice to not use the drug. The emerging picture concerning fluconazole treatment is that there is a need for more guidance for clinicians, through greater acknowledgement of the existing guidelines and acquisition of additional data on the drug.

One of the major limits of this study was the sample size. The Italian data originated from 38 NICUs and this limited data comparison.

Researchers in Italy have attempted to prioritize shared information on the care of extremely low birth weight babies [38,39], but the situation needs further developments. The collection of data on rates of infection by a centralized, international public body would permit transparency and homogeneous, accurate data on incidence, and this would, in turn, motivate centers to implement the most effective, evidence based practices.

The TINN project has already begun to improve the availability of information on fluconazole and ciprofloxacin, as also documented in published articles [20,40,41], and will provide additional clinical evidence in the early future.

## Conclusions

This national survey confirms the existence of very different approaches between NICUs towards the prevention and management of neonatal sepsis. The heterogeneity of the dosage schemes clearly demonstrates the lack of solid, acknowledged guidance, and probable lack of awareness of existing guidance.

The lack of hard evidence leads to different approaches and to the request for indications by third parties (i.e. scientific societies).

Common approaches are needed to standardize and update a national drug strategy for the prevention and treatment of sepsis in very low birth weight newborns. This can be achieved through collaborative initiatives aimed at setting up guidelines based on available data. The survey described in this article was a first step in acquiring data to stress the need for guidelines, to orient their contents towards the areas in greatest need of information, and to support neonatal societies in their creation. Formal international consensus statements, such as those that will be produced by the TINN network, will contribute to a more homogeneous and rational approach to neonatal sepsis prophylaxis and treatment in NICUs worldwide.

## Abbreviations

NICU: Neonatal intensive care unit; VLBW: Very low birth weight; CVC: Central venous catheter; TPN: Total parenteral nutrition.

## Competing interests

The authors declare that they have no competing interests.

## Authors’ contributions

CP participated in the creation and testing of the online questionnaire, in data cleaning, management, and analysis, and in drafting and revising the manuscript. MS carried out statistical data analyses and revised the manuscript. PM collaborated in conceiving and designing the data collection form and revised the manuscript. MB guided the creation of the questionnaire, the data analyses, and revised the manuscript. All authors read and approved the final manuscript.

## Pre-publication history

The pre-publication history for this paper can be accessed here:

http://www.biomedcentral.com/1471-2431/13/5/prepub

## Supplementary Material

Additional file 1 Likert scale calculations. Click here for file
