# Supplementary material for: The use of ciprofloxacin and fluconazole in Italian neonatal intensive care units: a nationwide survey
Source: BMC Pediatr. 2013 Jan 7;13:5. doi: 10.1186/1471-2431-13-5 (PMC3546886; doi:10.1186/1471-2431-13-5)
Supplement: Additional file 1 — Likert scale calculations. [file 1471-2431-13-5-S1.docx]

**Additional file 1 – Table.** Likert scale calculations

|  | N. respon-dents | Average importance given by all NICUs (from 1 to 5) | % of the NICUs that *do not use* the drug and who consider the factor very important | % of the NICUs that *use* the drug and who consider the factor very important | **P** |
| --- | --- | --- | --- | --- | --- |
| **CIPROFLOXACIN FOR TREATMENT** |  |  |  |  |  |
| **Ciprofloxacin should be reserved only for infections with multi-drug resistant microorganisms** | 32 | **4.2** | **77.8** | **100** | **N.A.** |
| **Uncertainty about safety of the use of ciprofloxacin in the newborn is great** | 32 | **4.2** | **77.8** | **80** | **0.6323** |
| **Additional efficacy studies of ciprofloxacin in neonatal bacterial sepsis are needed** | 31 | **4.0** | **76.9** | **40** | **0.2593** |
| **Uncertainty about pharmacokinetics of ciprofloxacin in the newborn is great** | 32 | **4.0** | **74.1** | **60** | **0.9191** |
| **Widespread use could lead to increased bacterial resistance** | 32 | **3.9** | **66.7** | **100** | **0.3264** |
| **Incidence of neonatal sepsis due to multi- drug resistant organisms is/is not high in your NICU** | 33 | **3.2** | **50** | **60** | **0.9413** |
| **Ciprofloxacin has a broad bacterial spectrum** | 32 | **2.9** | **40.7** | **20** | **0.7060** |
| **Ciprofloxacin has a good penetration in the cerebrospinal fluid** | 32 | **2.8** | **37** | **40** | **0.7060** |
| **Ciprofloxacin is less costly than other antibiotics used in the same indication** | 32 | **1.8** | **7.4** | **0** | **N.A.** |
| **FLUCONAZOLE FOR TREATMENT** |  |  |  |  |  |
| **Statement by Pediatric Societies (AAP) supporting the selection of one antifungal for treatment in neonates is needed** | 29 | **3.3** | **53.8** | **43.8** | **0.5884** |
| **Additional studies of efficacy in the perinatal population are needed** | 28 | **3.3** | **41.7** | **50** | **0.6617** |
| **Uncertainty about pharmacometrics of the other antifungal agents** | 29 | **2.9** | **30.8** | **43.8** | **0.7401** |
| **Uncertainty about safety of the other antifungal agents in the newborn is greater than for fluconazole** | 29 | **2.8** | **15.4** | **43.8** | **0.2155** |
| **The agent is less costly compared to other available antifungals** | 29 | **2.3** | **7.7** | **31.3** | **0.2728** |
| **FLUCONAZOLE FOR PROPHYLAXIS** |  |  |  |  |  |
| **Statement by Pediatric Societies (AAP) in support of routine use in a subset of newborns is needed** | 35 | **3.5** | **85.7** | **50** | **0.2002** |
| **Additional studies of efficacy of antifungal agent in the perinatal population is needed** | 36 | **3.3** | **71.4** | **48.3** | **0.4968** |
| **The incidence of candidiasis in your NICU is/is not high enough to justify prophylaxis** | 36 | **3.2** | **57.1** | **37.9** | **0.6182** |
| **The criteria of high-risk patients in whom prophylaxis should be attempted need clarification** | 36 | **3.1** | **71.4** | **34.5** | **0.1762** |
| **Uncertainty about pharmacometrics of the antifungal agent in the newborn is great** | 35 | **3.0** | **57.1** | **25** | **0.2366** |
| **The role of surveillance culture in identifying high-risk neonates needs clarification** | 35 | **2.9** | **42.9** | **25** | **0.6399** |
| **Uncertainty about safety of the antifungal agent in the newborn is great** | 36 | **2.8** | **85.7** | **20.7** | **0.0046 *** |
| **Widespread antifungal use could lead to increased antifungal resistance** | 35 | **2.7** | **50** | **27.6** | **0.5528** |
| **The agent is too costly** | 36 | **1.6** | **0** | **3.4** | **N.A.** |
